# Cost Sharing and Cost Shifting Mechanisms under a per Diem Payment System in a County of China

**DOI:** 10.3390/ijerph20032522

**Published:** 2023-01-31

**Authors:** Fengrong Liu, Jiayu Chen, Chaozhu Li, Fenghui Xu

**Affiliations:** 1 School of Public Policy & Management, Tsinghua University, Beijing 100084, China; 2 Jiyang College, Zhejiang Agriculture and Forestry University, Zhuji 311800, China; 3 School of Labor Economics, Capital University of Economics and Business, Beijing 100070, China

**Keywords:** per diem payment, supply-side cost sharing, cost shifting

## Abstract

Cost sharing and cost shifting mechanisms are of vital importance in a prospective payment system. This paper employed the difference-in-differences method to estimate the impacts of a per diem system with inverted-U-shape rates on medical costs and the length of stay based on data from a health insurance institution. The supply side cost sharing mechanism worked so that the new payment system significantly reduced medical costs by 17.59 percent while the average length of stay varied little. After further analyzing the mechanism, we found that heterogeneous effects emerged mainly due to the special rates design. The reform decreased the cases that incurred relatively high medical costs and lengths of stay. However, cost shifting existed so that physicians could be motivated to provide unnecessary services to the patients who should have been discharged before the average length of stay. Therefore, payment rates in the per diem system require a sophisticated design to constrain its distortion to medical service provision even though medical expenditures were successfully contained.

## 1. Introduction

It is widely acknowledged that the provider payment method which ameliorates incentive compatibility between supply- and demand-side, rather than price regulation, performs effectively in medical expenditure containment [1]. Typical retrospective payment systems, such as fee-for-service (FFS), remunerates almost any costs charged by physicians and has long been criticized for inducing medical service oversupply and price rising [2]. Under the retrospective payment, physicians can make more benefits by providing extra medical services to the patients and generating higher medical expenses. The phenomenon of roaring medical expenses caused by this type of moral hazard is common in all countries. At present, prospective payment or case-based payment systems, such as the diagnosis-related group payment (DRG), capitation, periodical payment, etc., are advocated worldwide as lots of practices and studies have proven their influences in decreasing medical costs [3,4,5].

In a prospective payment system, the payment rates are predetermined by diagnosis or demographic characteristics so that providers are involved in sharing the medical costs [6]. Surplus is generated to motivate cost containing when actual medical costs are less than the predetermined amount, while the proportion of medical expenses more than the prepaid amount will no longer be compensated but borne by the suppliers themselves. A prospective payment system thus unbinds the payment of the payers from the service costs determined by the suppliers, which limits the longstanding dominance of medical service providers in medical costs [7], and creates economic incentives for the suppliers to share and reduce medical costs [8]. Ellis and McGuire believe that the supply-side cost sharing in medical payment is more effective than that of the demand side [9]. There is a conflict between patient-side risk sharing and costs control. Although demand-side cost sharing limits the moral hazard of patients, it may also increase their financial burdens and cause the patients to drop insurance [10]. On the contrary, hospitals are more capable to bear risks than individuals so the supply-side cost sharing in the prepaid system is more likely to yield incentives. In addition, due to the dominant position of doctors in treatment plans and costs decisions, the effects of controlling medical expenses from the supply side could be stronger [11,12,13] and from the perspective of social value, citizens are resistant to lifting the level of individual out-of-pocket expenses.

The supply-side cost sharing mechanism can be affected by cost shifting. It is obvious that the prospective system still has incentive risks. Although it enables the expenditure responsibility to be passed from payers to suppliers, suppliers can further transfer risks. Researchers are concerned about doctors’ selection risks, including “creaming”, “skimping” and “dumping”. Creaming refers to providing too many medical services to patients with minor ailments, skimming refers to providing insufficient medical services to patients with high costs while dumping is refusing to treat patients with high costs [14]. Brekke et al. find that when the prepayment system is only applied to the social insurance, cost transfer emerges that providers would charge higher prices for private insurance and uninsured patients [15]. The cost transfer in the prospective system also has an impact on the medical market structure in the United States. Patients left earlier and sicker at discharge, and rehabilitation and nursing-care institutions grew rapidly [16,17].

A per diem system is considered to break the dichotomy between retrospective and prospective. On the one hand, remuneration per day is set ex ante to motivate physicians to decrease actual costs; on the other hand, providers are still dominant in patients’ length of stay (LOS) which further determines total costs [18]. As a result, not all the per diem payment could achieve the expected goal, additional incentives to curtail LOS and enhance accuracy would be required [19,20]. The per diem system combined with a per case payment including diagnosis groups or department classification has thus emerged in several countries, which adds the motivations to contain costs [21,22]. Furthermore, step-down payment rates tend to replace the flat rates to reduce unnecessary periods of hospitalization [23,24]. Degressive per diem rates are advocated but when and how to diminish the rates remains in doubt [25]. Only a limited number of studies have shed light on the mechanism of declining rates. The declining rates system may effectively shorten LOS but opposite empirical results have been provided to complicate its impact on medical expenditure [26,27].

This paper intends to illustrate the impacts of a per diem payment system on cost containment and LOS, and further explore the mechanisms of cost sharing and cost shifting. A difference-in-differences (DD) method was employed using health insurance data from 2012 to 2015 in township hospitals in a county in China. The data provided patient gender, age, main diagnosis and disease group, hospital and its category as independent variables to detect the policy impact on dependent variables such as medical expenditures and LOS. We believe that this study case could make marginal contributions to at least two aspects: the first one is the illustration of an easily implemented and effective payment system designed by officials in an average county in a developing country; the second is the emphasis to confront the adverse effects of rates design in a per diem system.

## 2. Institutional Background

The township hospitals in China are public institutions funded by the government. They are in the lowest tier of the three-tier hospital system and mainly provide services to the rural areas. Similar to most of the hospitals in China, all the public hospitals in the study county used to be reimbursed under an FFS system. Doctors employed in public hospitals in China are paid under the instruction of the salary system regulation for employees in public institutions released by Ministry of Human Resources and Social Security and Ministry of Finance in 2006 [28]. Their income package consists of three main parts: the fixed part set according to their position and their working experience; a performance related salary; and other subsidies. About 70 percent of a physician’s salary is linked to hospital income which is mainly decided by the medical service revenue of the department and government funding [3]. The salary decided by department and hospital revenue is estimated to account for approximately 30 percent [29] to 70 percent [30] in different literatures. The FFS system combined with the salary package further raises the risk of an induced demand of medical service and medical expenditure escalation.

According to official statistics, the average costs per patient in the county increased sharply by 16.38% from 2010 to 2012, resulting in an increase of more than 17 million yuan reimbursement by the insurance funds. Based on data from the National Bureau of Statistics, the inflation rate for health care (including pharmacy, materials and service for health) is only 4.6% during this period [31]. Cost sharing, total budget and direct or indirect supplier control measures are widely used to control medical expenses [32]. The county studied in this article had also executed various kinds of direct rewards or punishments measures to contain medical expenditure before the per diem payment reform. Unfortunately, those efforts were in vain. After this, they turned to other approaches including learning payment theories from publications and attending seminars to seek for better payment methods.

In 2014, health department officials in the study county crafted the per diem payment system associated with disease groups. It is a mixed payment method with disease grouping and per diem payment:

The first layer is disease grouping. Different from the complex “coarse-to-fine” procedures such as DRG grouping, the grouping rational here is a “coarse” one that depends on main diagnosis. Disease grouping starts from up to 18 different medical departments operated in the township hospitals of this county. For each department, the policy makers consulted medical and administrative experts to confirm patient volume and treatment features for different diseases thus determining about 50 disease groups. Disease groups range from only one to four in the departments based on the above principle. For example, the Department of Cardiology is the one which accepts a large number of inpatients and thus consists of four disease groups: hypertension, coronary heart disease, arrhythmia and others. Apparently, the first three groups are the most common diseases patients suffer in this department. Patients will be automatically assigned to the related group according to their main diagnosis. In the Department of Burn, there is only one group called diseases in the Department of Burn that includes burn injury, scald injury, electric shock and so on. In the Department of Surgery, the three disease groups are surgical diseases, non-surgical diseases and other diseases. It seems that the classification method can be flexible as treatment measures can also be the sorting standard. Historical health insurance data were analyzed and calculated in a per diem basis to determine the payment rates. Comparing with other payment innovations such as the Diagnosis-Intervention-Packet (DIP) and DRG mainly implemented in big cities in China, which contain hundreds of and even more than 10 thousand groups respectively [33,34], the coarse grouping method in this county is simple and more plausible for less developed areas to learn and rollout.

**Figure 1:**
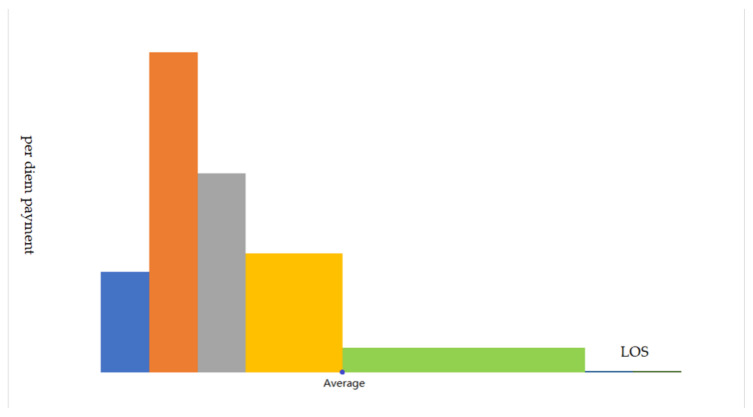
The inverted-U-shape per diem payment rates for a certain group.

The second layer is paid by the length of stay in various rates. For hospitalized patients, the length of stay reflects the severity of the disease or the complexity of the treatment. Therefore, taking a per diem payment into the system could make up the inaccuracy of the coarse grouping system to some extent. Distinguished from the flat rates or declining rates discussed in other literatures, the rates of the system here is in an inverted-U shape. It is more of a mapping of the actual distribution of medical costs’ progressivity than a deliberate design, such as declining rates. Figure 1 shows that the per diem payment first increases as LOS prolongs then drops to a moderate rate around the average LOS, and maintains at a very low rate until the last predetermined eligible day. The example group in Figure 1 consists of five stages which varies in rates from 21 yuan to the top of 272 yuan per day. Payment stages range from two to five across all the groups in accordance with their historical medical expenditure distribution patterns. This system stands out because the inverted-U-shape rates design is unique as it could not be found in other literatures as far as we know. The segmented payment standard means that the retrospective payment feature could be somewhat averted and could generate incentives to curtail the lengths of stay. Given the constraint of hospital beds and medical resources, the strategy of simply extending hospitalization time to obtain more medical income is no longer the optimal one. The inverted-U-shape pattern is expected to exert little distortion because it reflects the relationship between disease severity, service density and medical costs in reality. However, the effects of cost shifting are inevitable and the mechanism behind them demands further study.

## 3. Data and Methods

### 3.1. Data

De-identified health insurance data from 2012 to 2015 in the study township hospitals were applied in this paper. All the patients were covered by the same kind of health insurance where the reform took place. The records include demographic information (age and gender), hospital name, hospital category, main diagnosis, admission and discharge date, medical costs and disease grouping information for patients admitted after the payment reform. We simulated the grouping information for patients before the reform based on the main diagnosis. After excluding records relating to other payment system (2349 items), missing key information (482 items) and by mistakes (2 items), 69480 inpatient records were assessed in this paper. Among them, 38682 records under the former FFS system belonged to the control group whereas 30798 were in the reform group. Statistically, the medical costs in the reform group were 247.6 yuan lower than those in the control group, while the LOS was slightly less (0.007 days) in the reform group (Table 1).

### 3.2. Methods

Difference-in-differences is probably the most widely applied quasi-experimental method [35]. In this reform, 2 hospitals were first involved in March 2014, and other hospitals gradually participated until all of them were transferred to the new system in March 2015. Due to various times of intervention, the DD model here is with multi groups and multi periods [36], the model at the individual patient level can be:

(1) $${Y\;}_{ith}=\alpha_{0}+\alpha_{1}Reform_{iht}+\theta X_{ith}+\gamma_{h}+\delta_{t}+\varepsilon_{int}$$

where ${Y\;}_{ith}$ represents the explanation variables such as the medical costs or LOS incurred by patient *i* in hospital *h* at the time *t.* $Reform_{iht}$ equals to 1 when the record was generated under the per diem system with inverted-U-shape rates, otherwise, it equals 0. $X_{ith}$ contains control variables such as gender and age. $\gamma_{h}$ is the fixed effects of hospitals and $\delta_{t}$ is the fixed effects of time. We set the time fixed effects at the month level.

Next, a dynamic effects model is introduced to estimate trends before and after the reform [37]:

(2) $$\begin{array}{c} {Y\;}_{ith}=\alpha_{0}+\alpha_{1}Before_{i,t,h}^{-12}+\alpha_{2}Before_{i,t,h}^{-11}+\alpha_{3}Before_{i,t,h}^{-10}+\alpha_{4}Before_{i,t,h}^{-9}+\ldots +\alpha_{13}Before_{i,t,h}^{+1}\\ +\alpha_{14}Before_{i,t,h}^{+2}+\ldots +\alpha_{24}Before_{i,t,h}^{+12}+\theta X_{ith}+\gamma_{h}+\delta_{t}+\varepsilon_{int} \end{array}$$

where $Before_{i,t,h}^{-n}$ is 0 but equals 1 for admission in the *n*th month before the reform. Similarly, $After_{i,t,h}^{+n}$ is set 0 but equals to 1 for admission in the *n*th month after the implementation of the new system. Specially, $Before_{i,t,h}^{-12}$ is 1 for all the years that are 12 or more years before the reform and $After_{i,t,h}^{+12}$ equals 1 for all the years that are 12 or more years after it.

## 4. Results

### 4.1. Difference-in-Differences Estimates on the Reform Impacts

Table 2 displays the difference-in-differences estimates results of the reform impacts. Medical costs after the reform witnessed a significant fall by 17.59 percent. Considering that medical expenditures in these hospitals soared by 12.41 percent and 6.38 percent, respectively, in 2011 and 2012, the reform seemed to be practically effective in cost control.

For the discrete count variable, the length of inpatients stay, a poisson model was applied [38]. The estimates illustrate that reform impacts on LOS was statistically insignificant. It sparks the question that, from FFS to per diem payment, how can total medical expenditures plummet when the average LOS remained stable? Further studies should be conducted and we detected the heterogeneous response in Section 5.

### 4.2. Robustness Check with Dynamic Estimates

**Figure 2:**
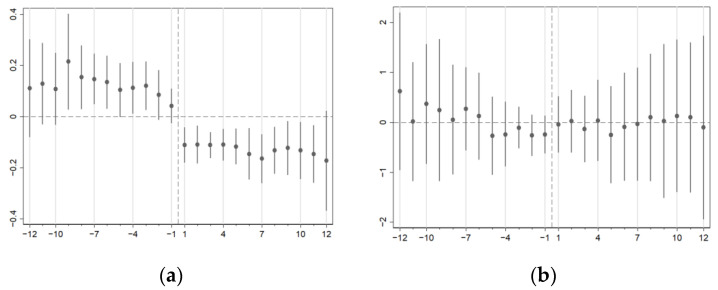
(**a**) Dynamic effects on medical costs; (**b**) Dynamic effects on LOS. Horizontal axis represents the month relative to the reform and vertical axis is the change in reform effects. Medical costs are in natural logarithm. We use poisson model for LOS as a discrete count variable. Standard errors cluster at hospital-time level.

Figure 2 is the dynamic effects of the reform on the dependent variables. With regard to medical costs, only some of the coefficients on the reform dummy were insignificantly different from zero (Figure 2a) while placebo tests proved that common trends existed (Table A1). We also found that medical costs reduced sharply right after the reform, and the first month with the new payment system experienced a significant 11.15 percent drop. In the following months, the decrease rates fluctuated and finally reached a maximum after 12 months.

In Figure 2b, there were no trends in difference between the control group and the reform group preceding the reform, which means these two groups had common trends of LOS. Meanwhile, the coefficients after the reform were also insignificant and consistent with the results in Table 2.

### 4.3. Heterogeneous Effects on Medical Costs and LOS

The mechanism behind the change is discussed in this section to provide an explanation to the heterogeneous responses and potential cost shifting.

**Figure 3:**
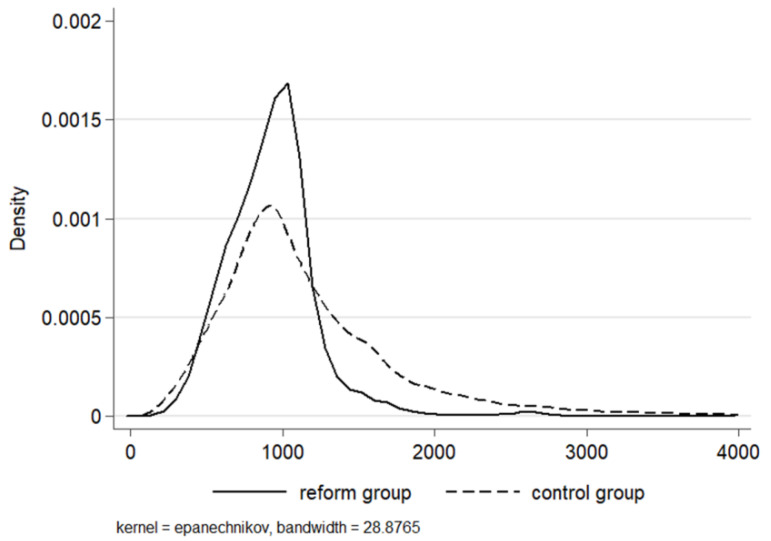
Distribution of the medical costs in the control and reform groups. To clearly display the density, a 95% winsorization of the high ends was processed.

As Figure 3 shows, the distribution of patient expenditures visibly changed after the reform. Generally speaking, medical costs concentrated more densely around 1000 yuan per case and reduced at both ends. Patients discharged with the bills below 500 and over 1200 yuan decreased significantly in the reform group. The distribution variation implied that heterogeneous effects may exist.

We then analyzed the heterogeneous changes of medical costs through the quantile difference-in-differences method (QDD). Overall, medical costs fell significantly at 1% level for all the quantiles in Table 3 and the impact of the reform amplified when moving to higher quantiles, with the coefficient continuously increasing from 10.72% (the 10th quantile) to 22.87% (the 90th quantile), which was more than doubled in size. Physicians are believed to have advantages in deciding treatment and the related costs, which could trigger supplier-induced demand to medical service overprovision especially under FFS [39,40].

**Figure 4:**
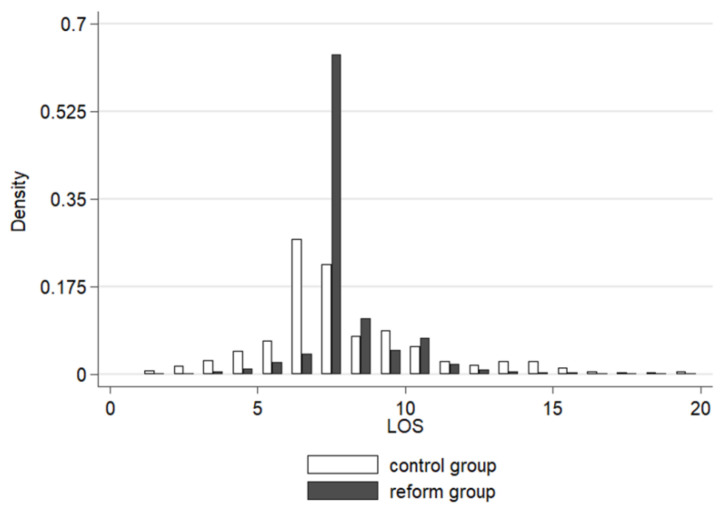
Distribution of the LOS in the control and reform groups. To clearly display the density, a 95% winsorization of the high ends was processed.

The main regression in Table 2 shows confusing results in that the average LOS remained almost unchanged after the reform. However, the distribution of LOS did change to a new pattern (Figure 4). In the control group, 6 days and 7 days accounted for 26.93 percent and 21.91 percent, respectively, and 82.09 percent of the inpatients spent 4 to 10 days in hospital. On the contrary, under the per diem system, patients with a 7-day-stay made up for more than half (63.88 percent) of the admissions. The length of 7, 8 (11.25 percent) and 10 (7.17%) days together contributed to a proportion of 82.3 percent. The structural transformation did not happen randomly but was associated closely with the inverted-U-shape rates. The 7th and 10th day were two vital thresholds in this payment system. In total, 47.73 percent of the disease groups reached their maximum marginal payment on the 7th day while 13.64 percent of those were on the 10th day. Apart from that, considering the longest eligible payment day of all the groups, the 10th day accounted for 22.73 percent.

Quantile difference-in-differences regression was also applied to test the structural variations of medical costs (Table 4). Signs of the coefficients were positive from the 10th to the 70th quantile and reversed to negative from the higher quantiles, which means that LOS expanded in low quantiles and decreased in the high ones. The impacts of the per diem system were more profound on both the ends where coefficients steadily reduced from the 10th quantile (17.95%) to the 70th quantile (3.18%), and enhanced again from the 80th quantile (−3.39%) to the 90th quantile (10.50%). Quantile estimates and the distribution of LOS (Figure 3) coincided so it converged at somewhere near the middle and led to the phenomenon of heterogeneous changes between the two ends.

The per diem system itself usually could not provide adequate incentives to cut down patients’ length of stay. Though the inverted-U-shape rates system was believed to fit actual distribution medical costs initially, it clearly imposed adverse effects on cases in low quantiles of LOS. The preset average LOS became a very strong motivation because marginal and average remuneration was the most until this day. Patients who should have been discharged before the average LOS tended to stay longer due to the new system. The payment rates should be further adjusted to tackle with the oversupply problem. Possible approaches may include decreasing the length before the average LOS and introducing mixed payment methods.

## 5. Discussions

The reform in this county turned out to be considerably effective in medical costs containment which cut off 17.59 percent of medical expenditures per case. The average LOS stayed unchanged but heterogeneous variations shed light on the effects of per diem rates on LOS and medical costs.

First, the changes of medical costs may be explained as declined supplier-induced provision under the new prospective-featured payment system. The new payment method did restrain the motivation of oversupply under FFS and, to some extent, squeezed out the inflation induced from the past. What is more, the effects did not distribute among all the patient groups. Higher-cost inpatients seemed to have been given more oversupply before the reform for reform effects enhanced in higher quantiles. However, we also noticed that suppliers under prospective payment can be motived to suppress medical expenditures at the cost of providing insufficient services to patients [9]. In a system with changing pre-payment rates, there is a risk that the cut of costs may result in inadequate services. In this case, policy makers should monitor service quality especially for those patients suffering severe diseases or facing relatively high medical costs.

Second, the main reason why the average LOS did not change was probably that the average LOS for each group before the reform was assigned the maximum marginal payment rates under the per diem payment system. The newly generated incentives were to discharge patients around the day with the highest per diem payment. As a result, though the number of the patients who stayed for a long period reduced, patients with short LOS decreased largely as well. Under the new system, a very small portion of patients were discharged within 5 days which means patients with minor diseases may be deliberately kept longer in hospital. Apparently, the inverted-U-shape rates of the per diem payment did not live up to the expectation of little distortion. Risks still remained as to whether appropriate services could be provided to all the patients. These results may verify the moral hazard model that physicians would supply more services to profitable patients [41], such as in the cases of the low quantiles of LOS. Even the per diem system with declining rates in Japan were facing similar adverse effects when its payment per day dropped largely after the average LOS [26]. Apart from the commonly discussed problems of insufficient service supply under the prospective payment system, attention should be drawn to monitor and deal with the potential oversupply problem as well. Finally, despite all the imperfections, a noticeable merit of the new payment method is that it was purposely tailored to the township’s hospitals in the case’s county. The coarse but inspiring payment reform greatly removed both the technical and acceptance barriers in an ordinary county in a developing country. First, the local officials made the reform optional instead of mandatory to the hospitals. Only hospitals that were ready and volunteered to join the new scheme would be paid based on per diem rates. Second, the coarse grouping method suited well to the reality of incomplete discharge records, a lack of clinical pathways [33], less expertise in coding [34] and doctors’ resistance to a complex grouping method in township hospitals. In addition, the functions of township hospitals in China meant that treatment density does not vary too much for they belong to the lowest tier of the three-tier hospital systems in China. They are mainly responsible for providing public health services, offering treatment to common health problems and providing rehabilitation services [42]. Third, the per diem payment rates were an approximate average of the history records which were inclined to at least maintain the hospital income even if the providers acted as before. Once the physicians were informed and comprehended that reducing unnecessary services could help them better adapt to the new system, they would change their behavior quickly. These three factors together made both the hospitals that joined the scheme at the beginning benefit from the reform soon and further encouraged more hospitals to participate and alter their treatment behaviors.

### Limitations

This study still has several limitations. First, the analysis of the medical quality change resulting from the reform mainly focused on whether necessary LOS were prescribed to all the patients and the potential moral hazard behind it. Due to the de-identified health insurance data, we were not able to process estimations of readmission, which could further clarify what really happened, especially to the patients with severe comorbidity. Second, we believe that social economic factors such as patient income or detailed disease information such as severity could also affect the outcomes of the reform. Unfortunately, these kinds of data were not available. The third limitation was related to whether cost shifting to other types of care outside the township hospitals were not included. We noticed that rehab facilities and nursery homes were frequently discussed when prospective payment was introduced into some other countries in the literature. However, here in China, these institutions are not very common and township hospitals are expected to receive patients discharged from larger hospitals who still need to rehabilitate.

## 6. Conclusions

The reform of the per diem payment in the study’s county significantly reduced the total medical expenditure by 17.59 percent. In the context of the continuous increase in the national hospitalization medical expenses, the average hospitalization expenditure of patients in this county not only decreased but even dropped below that before the reform. In a healthcare market where suppliers have the advantages of information and decision-making, a significant change in the level of medical expenses means that suppliers respond strongly to the implementation of the new policy. The payment reform impelled medical service providers to choose strategies to control medical expenses.

Based on quantile difference-in-differences estimates, we found that total medical expenditures and length of stay changed heterogeneously. The inverted-U-shape payment rates system induced physicians to oversupply to cases in the low quantiles after the reform while decreasing services to patients in the high quantiles. The results comport with the cost shifting hypothesis presented by Ellis [14] that the creaming and skimming mechanism works simultaneously. The inverted-U-shape payment turns out to be similar to the declining rates system where the predetermined average length of stay plays a critical role in affecting physicians’ behavior. Therefore, the per diem payment system requires delicate revision to the payment rates pattern and possible approaches may be taken to lower the risks of prescribing unnecessary stays in the hospitals or discharging the severe patients earlier. For instance, to avoid excess inpatient days, policy makers can set the LOS which enjoys the maximum payment rates lower than average LOS calculated from historical records. At the same time, FFS payment could be applied to the necessary stays which are longer than the current eligible payment days.

## Figures and Tables

**Table 1:** Statistics description.

	Whole		Control Group		Reform Group	
	Mean	S.D.	Mean	S.D.	Mean	S.D.
Gender	0.55	0.50	0.54	0.50	0.57	0.50
Age	58.74	14.12	56.89	15.38	61.05	11.97
LOS	7.56	2.72	7.60	3.34	7.53	1.64
Medical costs	1078.04	688.37	1187.79	815.71	940.19	446.30

**Table 2:** Impacts of the per diem payment with inverted-U-shape rates.

	Medical Costs	LOS
Reform	−0.1759 *** (0.0186)	0.0249 (0.0448)
Gender	−0.0135 *** (0.0050)	−0.0300 *** (0.0069)
Age	0.0138 *** (0.026)	−0.0061 *** (0.0019)
Age^2^	−0.0001 *** (0.0000)	−0.0001 *** (0.0000)
LOS	−0.0915 *** (0.0049)	
Disease variables	Yes	Yes
Hospital fixed effects	Yes	Yes
Time fixed effects	Yes	Yes
*n*	69,480	69,480
adj. *R*^2^	0.582	

The results are estimated according to Equation (1). Medical costs are in natural logarithm. We use poisson model for LOS is a discrete count variable. Standard errors are in parentheses, which cluster at hospital-time level. *** *p* < 0.01, ** *p* < 0.05, * *p* < 0.1.

**Table 3:** Quantile difference-in-differences estimates of medical costs.

	(1)	(2)	(3)	(4)	(5)	(6)	(7)	(8)	(9)	(10)
	OLS	Q10	Q20	Q30	Q40	Q50	Q60	Q70	Q80	Q90
Reform	−0.1759 ***	−0.1072 ***	−0.1359 ***	−0.1500 ***	−0.1667 ***	−0.1814 ***	−0.1975 ***	−0.2114 ***	−0.2250 ***	−0.2287 ***
	(0.0135)	(0.0109)	(0.0089)	(0.0079)	(0.0073)	(0.0068)	(0.0070)	(0.0070)	(0.0070)	(0.0091)
Control variables	YES	YES	YES	YES	YES	YES	YES	YES	YES	YES
Hospital fixed effects	YES	YES	YES	YES	YES	YES	YES	YES	YES	YES
Time fixed effects	YES	YES	YES	YES	YES	YES	YES	YES	YES	YES
*N*	69,480	69,480	69,480	69,480	69,480	69,480	69,480	69,480	69,480	69,480

Medical costs are in natural logarithm. Standard errors are in parentheses, which cluster at hospital-time level. *** *p* < 0.01, ** *p* < 0.05, * *p* < 0.1.

**Table 4:** Quantile difference-in-differences estimates of LOS.

	(1)	(2)	(3)	(4)	(5)	(6)	(7)	(8)	(9)	(10)
	Poisson	Q10	Q20	Q30	Q40	Q50	Q60	Q70	Q80	Q90
Reform	0.0249	0.1795 ***	0.1458 ***	0.1246 ***	0.1038 ***	0.0866 ***	0.0670 ***	0.0318 ***	−0.0339 ***	−0.1050 ***
	(0.0448)	(0.0060)	(0.0035)	(0.0037)	(0.0040)	(0.0049)	(0.0045)	(0.0059)	(0.0086)	(0.0120)
Control variables	YES	YES	YES	YES	YES	YES	YES	YES	YES	YES
Hospital fixed effects	YES	YES	YES	YES	YES	YES	YES	YES	YES	YES
Time fixed effects	YES	YES	YES	YES	YES	YES	YES	YES	YES	YES
*N*	69,480	69,480	69,480	69,480	69,480	69,480	69,480	69,480	69,480	69,480

We use poisson model for LOS is a discrete count variable. Standard errors are in parentheses, which cluster at hospital-time level. *** *p* < 0.01, ** *p* < 0.05, * *p* < 0.1.

## Data Availability

Data can be made available by contacting the corresponding author and with the approval of the study county officials.

## Appendix A

**Table A1:** Placebo tests of the effects on medical costs.

	Medical Costs
Reform	−0.0384 (0.0561)
Gender	−0.0198 ** (0.0083)
Age	0.0157 *** (0.0033)
Age^2^	−0.0001 *** (0.0000)
LOS	0.0888 *** (0.0056)
Control variables	Yes
Hospital fixed effects	Yes
Time fixed effects	Yes
*N*	28,021
adj. *R*^2^	0.609

Medical costs are in natural logarithm. Standard errors are in parentheses, which cluster at hospital-time level. *** *p* < 0.01, ** *p* < 0.05, * *p* < 0.1.
